# Natural killer cells in herpesvirus infections

**DOI:** 10.12688/f1000research.11197.1

**Published:** 2017-07-26

**Authors:** Christian Münz, Obinna Chijioke

**Affiliations:** 1 Viral Immunobiology, Institute of Experimental Immunology, University of Zurich, Winterthurerstrasse, Zurich, Switzerland; 2 Institute of Pathology and Molecular Pathology, University Hospital Zurich, Schmelzbergstrasse, Zurich, Switzerland

**Keywords:** natural killer cells, protection, effector, subsets, herpes simplex virus

## Abstract

Natural killer (NK) cells are potent innate cytotoxic lymphocytes for the destruction of infected and transformed cells. Although they were originally considered to be ready-made assassins after their hematopoietic development, it has recently become clear that their activity is regulated by mechanisms such as repertoire composition, licensing, priming, and adaptive memory-like differentiation. Some of these mechanisms are influenced by infectious disease agents, including herpesviruses. In this review, we will compare expansion, stimulation, and effector functions of NK cell populations after infections with β- and γ _1_-herpesviruses because, though closely related, these pathogens seem to drive completely opposite NK cell responses. The discussed findings suggest that different NK cell subsets expand and perform protective functions during infectious diseases and might be used diagnostically to predict resistance to the causative pathogens as well as treat them by adoptive transfer of the respective populations.

## Introduction

Natural killer (NK) cells are innate lymphocytes that have been initially identified by their ability to kill tumor or infected cells without prior activation ^1– 3^. Their target cell recognition is composed of signals that they receive from germ-line encoded activating and inhibitory receptors, and the net outcome of these interactions leads to recognizing or passing over respective cellular targets ^4^. A surplus of activating signals can be achieved either by loss of inhibition, named “missing-self” recognition, or by upregulation of stimulation, named “altered-self” recognition ^5^. Inhibitory NK cell receptors recognize primarily classical and non-classical major histocompatibility complex (MHC) class I molecules, and thereby “missing-self” recognition counterbalances cytotoxic CD8 ^+^ T-cell recognition restricted by pathogen- or tumor-induced loss of MHC class I ^6^. While CD94/NKG2A recognizes signal peptides of other MHC class I molecules on non-classical human HLA-E or mouse H2-Qa1 molecules for its inhibition, killer immunoglobulin-like receptors (KIRs) in humans and Ly49 molecules in mice can distinguish polymorphic classical MHC class I molecules. In contrast, activating signal recognition is less well understood and more diverse but requires—with the exception of dominant triggering by opsonizing antibody binding to CD16 on NK cells—the stimulation of at least two activating receptors to unleash NK cell responses ^4^. These are often referred to as main activating receptors, like NKG2D, or the natural cytotoxicity receptors (NCRs) NKp30, NKp46, and NKp44 plus co-receptors that need to be engaged, including signaling lymphocyte-activating molecules (SLAMs) 2B4 and NTB-A, as well as DNAX accessory molecule-1 (DNAM-1). Their ligands are diverse, including MHC class I-like molecules for NKG2D, B7 family members like B7-H6 for NKp30, CD48 for 2B4, and poliovirus receptor (PVR) or Nectin-2 for DNAM-1. Some of these ligands are upregulated upon cellular stress, like uncontrolled proliferation and associated DNA damage responses ^7^. Thus, NK cells can integrate a diversity of clues from the surface of transformed and infected cells for their recognition.

Apart from this multitude of receptors, NK cell activity is regulated by at least four additional mechanisms, namely the NK cell repertoire, NK cell activation by cytokines or priming, adaptive or memory-like NK cell differentiation, and NK cell licensing. The NK cell repertoire is composed of up to 30,000 subpopulations ^8^. These subpopulations differ among each other with respect to inhibitory and activating receptor expression. Inhibitory receptor distribution is determined mainly by the respective genotype of the individual ^8– 11^. For example, KIRs are highly polymorphic and NK cell subsets with no, one, or multiple KIRs exist in any given individual. In contrast, activating NK cell receptor expression differs between monozygotic twins and therefore seems to be regulated by environmental factors ^8^. This variability amounts to up to 30,000 different NK cell subsets in the NK cell repertoire of any given human individual ^8^. Furthermore, cytokines—mainly interleukin-2 (IL-2), IL-12, IL-15, IL-18, and type I interferon—augment NK cell function and activate NK cells in secondary lymphoid tissues ^12– 14^. This activation is often mediated by dendritic cells (DCs). Furthermore, the NK cell compartment contains, to a variable degree, adaptive or memory-like NK cells. These hyper-reactive and long-lived NK cells have been described after certain, mostly viral antigen encounters and are able to mount stronger protective responses upon re-encounter of the same pathogen and sometimes even antigen ^15, 16^. These adaptive NK cells will be discussed in more detail in the context of persistent infections with β-herpesviruses below. Finally, NK cell reactivity is also adjusted to its environment by a process called licensing ^9, 17, 18^. Licensing describes a process by which NK cells, which carry inhibitory receptors that are engaged by healthy cells, are more reactive during “missing-self” recognition. It has been suggested that in the absence of an inhibitory signal the continuous stimulation of activating receptors leads to disarming of NK cells, attenuating their activity. Thus, multiple NK cell receptors as well as the composition of the NK cell compartment with licensed, cytokine-activated, adaptive, and different receptor-expressing cells contribute to the reactivity of these innate cytotoxic lymphocytes. All of these regulatory mechanisms contribute to their role during herpesvirus infections.

## Protection from herpesvirus infections by natural killer cells

Herpesviruses are double-stranded enveloped DNA viruses that establish persistent infections ^19^. They are exquisitely adapted to their host species and its immune control. Indeed, the first description of a primary immunodeficiency in NK cells characterized a patient who had uncontrolled herpesvirus infections ^20, 21^. Herpesviruses fall into the groups of neurotrophic α-herpesviruses, at least in part myelotrophic β-herpesviruses, and lymphotrophic γ-herpesviruses. The paradigmatic representatives of these three subclasses in humans are the herpes simplex virus (HSV), the human cytomegalovirus (HCMV), and the Epstein-Barr virus (EBV), respectively. Although the basic composition of these viruses is very similar, the influence on the NK cell compartment and their restriction by it during infection are very different. With respect to phenotype, recurrent HSV2 infection does not change the NK cell composition ^22^. In contrast, β-herpesvirus infection with HCMV has become the paradigm of human adaptive NK cell differentiation with an accumulation of terminally differentiated NK cells ^23^. Finally, the γ-herpesvirus EBV expands early differentiated NK cells during primary infection ^24^. In addition to the phenotypic differences in the NK cell responses to different herpesviruses, their dependency on NK cell-mediated immune control differs significantly. Even though in the above-mentioned GATA2-deficient patient ^20, 21^ recurrent α-herpesvirus infections were observed, the protection from HSV infection by NK cells in mouse models is still controversial and might depend on the site of infection as well as the investigated mouse strain ^25– 27^. In contrast, immune control of β-herpesvirus infection (HCMV) was also compromised in the original NK cell-deficient indicator patient and HCMV is used as the paradigmatic viral infection to investigate protective NK cell responses in mice ^28^. Finally, NK cells control lytic replication by the human γ-herpesvirus EBV and protect mice with human immune system components from enhanced tumor formation by this virus ^29^. Along these lines, individuals with MCM4 mutations and diminished NK cell compartments suffer from uncontrolled EBV infection ^30^. In contrast, the mouse γ _2_-herpesvirus MHV-68 is not affected by NK cell depletion during its infection in mice ^31^. Therefore, in the following sections, we will discuss cytomegalovirus and EBV as the two extremes for the remodeling of the NK cell compartments, although both of these viruses are controlled by NK cells.

## Natural killer cell phenotypes during herpesvirus infections

**Figure 1.**
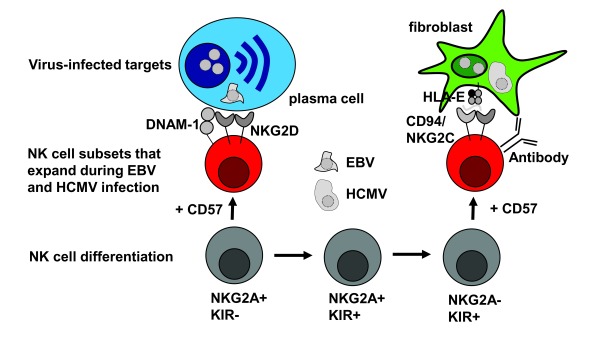
Differentiation and stimulation of human natural killer (NK) cells during Epstein-Barr virus (EBV) and human cytomegalovirus (HCMV) infection. Human NK cells differentiate with acquisition of killer immunoglobulin-like receptor (KIR) expression and lose NKG2A expression upon terminal differentiation. Expression of the senescence marker CD57 removes NK cell subpopulations from this differentiation. Lytically EBV-replicating plasma cells are preferentially recognized by early differentiated NK cells via their NKG2D and DNAX accessory molecule-1 (DNAM-1) receptors, while HCMV-infected cells expand terminally differentiated NK cells via CD94/NKG2C stimulation by HCMV peptide-presenting human leukocyte antigen (HLA)-E molecules. HCMV-infected cells are targeted by these terminally differentiated NK cells after antibody opsonization.

The remodeling of the NK cell phenotype by herpesvirus infections has been best described for human NK cells ^23, 24^. Human NK cells are thought to originate from the bone marrow as CD56 ^bright^CD16 ^−^ cells with homing markers (CCR7 and CD62L) for secondary lymphoid tissues ^32, 33^. There, they can acquire cytotoxic function and KIRs upon activation by DC-derived cytokines ^14^. It is thought that during this differentiation ( Figure 1), they acquire more and more KIRs and eventually lose expression of the inhibitory CD94/NKG2A receptor ^34^. CD94/NKG2A expression can be reacquired in inflammatory environments through IL-12-dependent induction ^35^. However, all of these intermediate NK cell populations can differentiate from proliferating to CD57-positive senescent NK cells and thereby arrest in their differentiation ^34^.

During persistent HCMV infection of healthy virus carriers, after HCMV reactivation in bone marrow transplant patients, in HCMV-infected children, and in HCMV-infected individuals with asymptomatic co-infections ^23, 36– 38^, terminally differentiated NK cells (phenotypically CD56 ^dim^CD16 ^+^KIR ^+^LIR1 ^+^NKG2C ^+^CD57 ^+^Bcl-2 ^+^NKG2A ^-^NKp46 ^low^NKp30 ^low^CD161 ^low^CD7 ^low^Tim-3 ^low^PZLF ^low^Syk ^low^Eat-2 ^low^DAB2 ^low^Helios ^low^FcεR1γ ^low^CD2 ^high^) with many epigenetically silenced gene loci accumulate ( Figure 1) ^39– 42^. These terminally differentiated NK cells might be generated by two synergistic mechanisms. The activating CD94/NKG2C receptor on this NK cell subset can engage HCMV peptide-presenting HLA-E molecules on infected fibroblasts ( Figure 1) ^43– 46^. However, these expand the above-characterized NKG2C ^+^ NK cell subset usually only in the presence of monokines like IL-15 and IL-12, which have been shown to be provided by bystander monocytes ^43, 44^. In fact, NKG2C-dependent HLA-E recognition might not be strictly required for the expansion of terminally differentiated NK cells during HCMV infection because the 4% of the human population that lack NKG2C expand NK cells of similar phenotype during chronic HCMV infection ^41, 47^. Instead, cytokines might be sufficient for the expansion of the respective NK cells, and the accumulation of this NK cell subset seems to be further augmented by co-infections with hantavirus, chikungunya virus, HIV, and hepatitis virus ^36, 48– 52^, which might enhance NK cell-differentiating cytokine expression. It is tempting to speculate that HCMV infection of the myeloid lineage favors the production of NK cell-differentiating cytokines that lead to the accumulation of NKG2C ^+^ NK cells.

B lymphotropic EBV also causes NK cell expansion during primary infection ^53– 56^. In particular, CD56 ^dim^CD16 ^+/−^KIR ^−^CD57 ^-^ NK cells proliferated during acute infectious mononucleosis (IM) ( Figure 1), and the frequency of this proliferating NK cell subset correlated with viral loads ^24^. The expansion of these early differentiated NK cells persists for at least 6 months ^24, 57, 58^, but they stop proliferating during this time period and acquire the senescence marker CD57 ^24, 58^. Interestingly, newborns carry a high frequency of these early differentiated KIR ^-^ NK cells, which are progressively lost during the first decade of life ^24^. This differentiation might be induced by other pathogens and HCMV could contribute significantly to this loss of early differentiated NK cells. Thus, the β-herpesvirus HCMV and the γ-herpesvirus EBV drive the expansion of completely different NK cell phenotypes, and it remains to be seen whether one virus thereby influences the immune control of the other.

## Effector functions of natural killer cells during herpesvirus infections

Indeed, this early differentiated NK cell phenotype in children correlates with a higher frequency of asymptomatic primary EBV infection, whereas delay of initial infection with this γ-herpesvirus leads, increasingly with age, to a higher likelihood of having immunopathologic symptoms of lymphadenopathy, fever and fatigue, which are caused by massive CD8 ^+^ T-cell expansion and are collectively called IM ^59^. Indeed, African children often suffer from the same high viral loads as patients with IM, but the CD8 ^+^ T-cell expansion of the former is less pronounced and thus no disease is experienced ^60^. It is tempting to speculate that despite high viral loads early differentiated innate lymphocytes, including NK cells, primarily deal with the infection, curbing CD8 ^+^ T-cell lymphocytosis. Indeed, such a protective function of NK cells during primary EBV infection was recently documented ^29^. In mice with reconstituted human immune system components, early differentiated NK cells predominate ^61^. Infection with EBV led to the expansion of these NK cells, starting 3 weeks after infection ^29^. This time point coincides with the time point at which lytic replication of EBV can be detected in this *in vivo* model, as judged by comparing wild-type with lytic replication-deficient BZLF1 ^-^ EBV ^62^. Depletion of NK cells with an antibody directed against NKp46 leads to elevated viral loads, starting at 3 weeks after infection ^29^. Viral load is elevated 10-fold in total blood and 100-fold in the serum ^29^. Only lytic EBV infection is affected because viral load of BZLF1 ^-^ EBV did not increase upon NK cell depletion ^29^. In good agreement with these findings, NK cells primarily recognize lytically EBV-infected targets ^24, 63^ and preferentially the early differentiated KIR ^-^ NK cells degranulate ^24^. This recognition has been suggested to be mediated by NKG2D and DNAM-1 ( Figure 1) ^63^. Interestingly, patients with deficiency in a magnesium transporter (MAGT1), resulting in diminished surface expression of NKG2D on NK and T cells, suffer from EBV-associated lymphoproliferations ^64^. In the absence of NK cells, EBV-infected mice with reconstituted human immune system components develop mostly monoclonal lymphoproliferations as well as CD8 ^+^ T-cell lymphocytosis, splenomegaly, and cytokinemia, which are hallmarks of IM ^29^. These studies suggest that NK cells—in particular, early differentiated KIR ^-^ NK cells—restrict lytic EBV replication and could explain the risk of adolescents for IM.

In contrast, the function of the terminally differentiated NKG2C ^+^ NK cells during HCMV infection is less clear. During mouse cytomegalovirus (MCMV) infection of C57BL/6 mice, Ly49H ^+^ NK cells preferentially expand and directly bind with their Ly49H receptor to the MCMV m157 protein on the surface of infected cells ^65, 66^. NK cells are indeed required for efficient immune control of MCMV infection ^67, 68^, and Ly49H ^+^ antigen-experienced NK cells control MCMV infection better than other subsets ^15^. Even though NKG2C ^+^ and NKG2C ^-^ human NK cells might represent the counterparts of the recently described Ly49H ^+^ and Ly49H ^-^ mouse NK cells, which acquire their adaptive functional superiority by either receptor- or cytokine-mediated stimulation, respectively ^69^, it has remained difficult to demonstrate a protective function for the NK cell expansions during HCMV infection. Although these terminally differentiated NKG2C ^+^ NK cells more readily produce cytokines in response to HCMV-infected cells ^70, 71^ and their frequency correlates with protection from HCMV disease after kidney transplantation ^72^, they seem to clear infected targets only after antibody-mediated opsonization by antibody-dependent cellular cytotoxicity ( Figure 1) ^73, 74^. This would argue for a protective role of these accumulating NK cells rather late during the infection, when HCMV-specific antibodies have already formed. Indeed, during hantavirus co-infection, the enhanced functionality of these NKG2C ^+^ NK cells was suggested to cause immunopathology by promoting vascular leakage via uninfected endothelial cell killing ^75^. Thus, KIR ^-^, NKG2C ^+^, and Ly49H ^+^ NK cell subpopulations expand and persist for several months during EBV, HCMV, and MCMV infection, but although protection of the respective NK cell subset during EBV and MCMV infection has been demonstrated, this remains less clear for HCMV infection.

## Conclusions

The extent of the complexity of the human NK cell compartment with up to 30,000 distinct subpopulations has only recently been appreciated ^8^. As discussed above, certain pathogens, exemplified in this review by the human herpesviruses HCMV and EBV, seem to drive expansions of distinct NK cell subsets, which then persist at elevated frequencies for months ^23, 24^. The protective features of these expanded NK cell populations are beginning to emerge ^29, 74^, as are how their expansion can be stimulated ^44^. Thus, it might be possible not only to use the NK cell phenotype as a predictor of immune control against these specific pathogens but also to adoptively transfer or stimulate these NK cell subsets in patients with diminished immune control of the respective viruses, starting with HCMV and EBV. However, in order to narrow the NK cell phenotype that might be required for clinical benefit, the receptor interactions and effector functions that mediate protection need to be better defined in the future.

## Abbreviations

DC, dendritic cell; DNAM-1, DNAX accessory molecule-1; EBV, Epstein-Barr virus; HCMV, human cytomegalovirus; HSV, herpes simplex virus; IL, interleukin; IM, infectious mononucleosis; KIR, killer immunoglobulin-like receptor; MCMV, mouse cytomegalovirus; MHC, major histocompatibility complex; NK, natural killer.
